# Quantification of transcriptome changes to investigate the role of glucocorticoid receptor-RNA binding during dexamethasone treatment

**DOI:** 10.1186/s13104-023-06446-4

**Published:** 2023-08-22

**Authors:** Nickolaus C. Lammer, Mary A. Allen, Robert T. Batey, Deborah S. Wuttke

**Affiliations:** 1 Department of Biochemistry, University of Colorado at Boulder, Campus Box 596, Boulder, CO 80309-0596 USA; 2 BioFrontiers Institute, University of Colorado at Boulder, Campus Box 596, Boulder, CO 80309-0596 USA

**Keywords:** Transcription factor, Glucocorticoid receptor, RNA binding, RNA sequencing, Transcriptomics, 4-thiouridine, NR3C1

## Abstract

**Objectives:**

The glucocorticoid receptor (GR) is a well-studied, ligand-activated transcription factor and a common target of anti-inflammatory treatments. Recently, several studies have drawn attention the effects of binding of GR to RNA rather than DNA and the potential implications of this activity for GR function. The objective of our study was to further characterize the relationship between GR function and RNA binding by measuring changes in the glucocorticoid-driven transcriptome in the presence of a GR mutant that exhibited reduced RNA affinity.

**Data description:**

GR was activated in three cell lines containing GR constructs (GR-HaloTag). One of the cell lines contained a wild-type GR-HaloTag. Another contained GR-HaloTag with a mutation that reduced RNA affinity and slightly reduced DNA affinity. The third cell line contained GR-HaloTag with a mutation that only slightly reduced DNA affinity. All three cell lines were treated with dexamethasone, a GR agonist. RNA-seq samples were collected every hour for 3 h. Moreover, transcriptome quantification was accomplished via labeling of RNAs transcribed in the final hour of dexamethasone treatment using 4-thiouridine. These labeled RNAs were then purified and sequenced. This data set is the first of its kind for GR and contains valuable insights into the function of RNA binding by GR.

## Objective

The glucocorticoid receptor (GR) is a ubiquitous transcription factor regulating many genes associated with stress response, inflammation, and apoptosis [1–5]. In medicine, its anti-inflammatory properties are commonly induced using the synthetic glucocorticoid dexamethasone [6]. Because of its important role in controlling inflammation, the regulation of GR has been studied in great detail. In the absence of glucocorticoids, GR is mostly found in the cytoplasm in its inactive form. Once glucocorticoids diffuse into the cell, they can bind to GR and initiate rapid nuclear translocation and subsequent regulation of downstream genes [3, 6–8]. Direct gene activation by GR typically happens through dimerization on sequence-specific DNA elements, and thus most studies of GR-mediated gene regulation have focused on this interaction [9–13]. However, several groups identified an additional, unexpected biochemical activity of direct RNA binding by GR to a common underlying motif of hairpin RNAs [2, 14–16]. Further, in vitro studies revealed structure-specific binding to RNA that is competitive with DNA [17]. The implications of these findings are poised to change how we think about both GR biology and, to a greater extent, the regulation of many transcription factors, by adding another layer to GR’s complex regulatory pathway. The objective of this data set is to better define the relationship between GR-RNA binding and GR transcriptional action through the quantification of the dexamethasone-driven transcriptome in the context of wild-type (wt) and RNA-binding deficient GR.

## Data description

To measure the effect RNA binding by GR has on the GR-regulated transcriptome, we performed RNA sequencing of 4-thiouridine-labeled RNAs (4sU-seq, Data set 1, Table 1) after dexamethasone treatment in cells expressing a separation-of-function GR mutant. We employed U2OS cells stably expressing wt GR-HaloTag or a K492A mutant of GR that has an 11-fold reduction in RNA affinity based on in vitro binding assays [17]. Due to a moderate reduction in DNA affinity in this mutant, we also used cells expressing an R470A GR mutant that exhibits similar reduction in DNA affinity but maintains RNA affinity. As U2OS cells express low levels of endogenous GR and show impaired glucocorticoid response, we can compare transcriptional responses to dexamethasone between wt GR and our mutant to attribute the function of GR-RNA binding to known regulatory pathways. After stable integration of the GR-HaloTag constructs, cells were sorted using FACS and sorted cell fractions were selected to match GR-HaloTag expression between cell lines by immunofluorescence. This sorting was done to minimize the impact of GR-HaloTag abundance on differential dexamethasone response between cell lines. After selecting cell lines with similar GR-HaloTag expression, cells were incubated with 100 nM dexamethasone for 0, 1, 2, or 3 h. Zero-hour dexamethasone cells received an equivalent volume of ethanol (0.01%). During the final hour of dexamethasone treatment, cells were given 200 µM 4sU to label recently transcribed RNAs. Zero-hour dexamethasone cells treated with 200 µM 4sU for 1 h immediately after adding ethanol. At the end of treatment, cells were lysed with TRIzol and RNAs were purified. 4sU-labeled RNAs were then biotinylated and isolated using magnetic streptavidin beads. A detailed 4sU-seq protocol can be found in Data file 1, Table 1.

Libraries for were prepared using the KAPA RNA HyperPrep kit with RiboErase. The dual index adapters included unique molecular identifiers (UMIs). Library sizes were about 330 bp and they were sequenced for 2 × 150 bp paired-end reads on an Illumina NovaSEQ 6000 by the University of Colorado School of Medicine Genomics and Microarray Core Facility. Reads were tagged with UMIs using UMI-tools (v1.1.2) and trimmed using Trim Galore (v0.6.6) with the following parameters: --2colour 20, --paired [18, 19]. Trimmed reads were then aligned to the hg38 genome assembly using STAR (v2.7.3a) and deduplicated with UMI-tools (v1.1.2) using the following parameters: --paired, --unpaired-reads discard, --chimeric-pairs discard [18, 20]. FeatureCounts (Rsubread v2.0.1, R v4.0.3) was used to count deduplicated read coverage over exons (Data file 2, Table 1) [21]. Quality control analyses were performed with FastQC on trimmed reads and RSeQC on aligned reads before deduplication and packaged together with MultiQC (Data file 3, Table 1) [22–24]. All scripts used for data processing, including options and flags used at each step, can be found on GitHub (Data set 2, Table 1).

**Table 1:** Overview of data files/data sets

Label	Name of data file/data set	File types (file extension)	Data repository and identifier (DOI or accession number)
Data set 1	Effect of reduced glucocorticoid receptor-RNA affinity on dexamethasone treatment	FASTQ files (fastq.gz)	NCBI GEO (https://identifiers.org/geo:GSE216337) [25]
Data file 1	4sU-seq protocol	PDF (.pdf)	Zenodo (10.5281/zenodo.7349186) [26]
Data file 2	Count matrix of deduplicated reads over exons	CSV (.csv)	NCBI GEO (https://identifiers.org/geo:GSE216337) [25]
Data file 3	MultiQC quality report	HTML (.html)	Zenodo (10.5281/zenodo.7269135) [27]
Data set 2	Data processing scripts	Bash script (.sh), R code (.r)	Zenodo (10.5281/zenodo.7349062) [28]

## Limitations

To meaningfully compare transcriptomic changes between the different GR-expressing cell lines, it was crucial to first match GR expression between cell lines. We sorted cells and selected fractions that had the same nuclear signal after dexamethasone treatment based on immunofluorescence. Nuclear signal was used to match the amount of active GR between cell lines, but transcript expression of GR is higher in the mutant lines.

## Data Availability

The data described in this Data note can be freely and openly accessed on NCBI GEO under GSE216337 (https://identifiers.org/geo:GSE216337). The detailed protocol for 4sU-seq can be accessed through Zenodo (10.5281/zenodo.7349186). Quality assessment is available through Zenodo (10.5281/zenodo.7269135). Data processing scripts are available through GitHub (via Zenodo, 10.5281/zenodo.7349062). Please see Table 1 and references [25–28] for details and links to the data.

## Abbreviations

GR: glucocorticoid receptor
4sU: 4-thiouridine
wt: wild-type
UMI: unique molecular identifier
